# Effect of an Intensive, Integrated Telehealth Intervention on Glycemic Control in Children and Adolescents With Type 1 Diabetes Using Continuous Glucose Monitoring: A Randomized, Crossover Trial

**DOI:** 10.1155/pedi/7261998

**Published:** 2025-09-27

**Authors:** Asma Deeb, Lubna Eldeeb, Shaker Suliman, Deepti Chaturvedi, Mary Tomy, Ghada Alkahlout, Reem Hassan Beck, Nabras Al Qahtani

**Affiliations:** ^1^Paediatric Endocrine Division, Sheikh Shakhbout Medical City P.O. Box 11001, Abu Dhabi, UAE; ^2^Faculty of Health and Science, Khalifa University, Hadbat Al Za'Faranah - Zone 1, Abu Dhabi, UAE; ^3^Clinical Trial Unit, Sheikh Shakhbout Medical City P.O. Box 11001, Abu Dhabi, UAE

**Keywords:** adolescents, continuous glucose monitoring, intervention, randomized crossover trial, teleconsultation

## Abstract

**Aim:** To examine the impact of adding an intensive, integrated telehealth intervention on glycemic control in children and adolescents with type 1 diabetes using continuous glucose monitoring (CGM) and multiple daily injections (MDIs) of insulin.

**Materials and Methods:** In this randomized, two-period crossover trial conducted between May 2023 and June 2024, 105 children and adolescents with type 1 diabetes using FreeStyle Libre 2 CGM were randomized to receive intensive telehealth weekly over 12 weeks first followed by routine care (*n* = 50) or routine care over 12 weeks first followed by intensive telehealth weekly (*n* = 55), with a 2-week washout. Intensive telehealth was intensified follow-up with weekly teleconsultation (20 min, by telephone) and digital support from a trained diabetes educator delivering structured support, including review of the latest ambulatory glucose profile. The primary outcome measures were HbA1c and GCM metrics.

**Results:** The average (SD) age of the study cohort (*n* = 105) was 11.8 (4.2) years, 48.6% were female, with an average diabetes duration of 3.5 (3.0) years and suboptimally controlled diabetes in terms of HbA1c levels (9.4 (1.6) %, target < 6.5%), and other 14-day CGM metrics. Compared with routine care, intensified follow-up with weekly intensive telehealth was associated with a decrease in HbA1c (−0.29 (0.60) %, 95%CIs −0.41 to −0.17, *p*  < 0.001), significantly increased time in range (TIR), and decreased time above range (TAR), average glucose level, glucose variability, glucose management indicator (GMI), and frequency of low glucose events. Teleconsultation did not affect time below range (TBR), which was already within target.

**Conclusion:** This randomized, controlled, and crossover study shows that intensified follow-up with a weekly telehealth intervention results in small but significant improvements in glycemic control metrics in children and adolescents. The clinical impact of these changes requires prospective study.

## 1. Introduction

Managing children and adolescents with type 1 diabetes has always been challenging, and achieving adequate glycemic control in this age group to reduce the long-term complications of the disease is difficult. Although thresholds for target HbA1c have changed over time [1], longitudinal study and registry data [2–4] consistently show that, regardless of definition, under a half—and usually closer to 20%—of adolescents with type 1 diabetes achieve adequate glycemic control.

These challenges persist despite advances in technologies available to patients and families living with diabetes, including insulin pumps, insulin analogs, and continuous glucose monitoring (CGM) systems. CGM provides near real-time information on blood glucose levels and trends, allowing rapid adjustment of insulin. Systematic reviews and meta-analyses show that use of CGM systems by children and adolescents positively impacts glycemic control endpoints, such as HbA1c levels and risk of severe hypoglycemic events, both with and without hybrid closed loop technology [5, 6]. Nevertheless, the persistent difficulty in achieving adequate glycemic control in this age group, despite the use of advanced technologies [7, 8], mandates interventions that improve outcomes in this at-risk group. Randomized controlled trials (RCTs) in adults with type 1 and type 2 diabetes have shown that telemedicine significantly decreases HbA1c compared with usual care [9], but data in children and adolescents are equivocal. Structured telemedicine care using video consultations may be a feasible, efficient, and cost-effective option to enhance diabetes care and improve glycemic control in young people struggling to attain recommended targets.

We hypothesized that augmenting CGM with intensive follow-up with a structured telehealth intervention that integrates teleconsultation, tele-education, and digital data support (online CGM analysis) might improve diabetes control. To achieve this, we performed a randomized, controlled, crossover study to examine the impact of adding teleconsultation and digital data support to CGM use on glycemic control in children and adolescents with type 1 diabetes taking multiple daily injections (MDIs) of insulin.

## 2. Subjects, Materials, and Methods

This trial is reported according to CONSORT 2010 guidelines with an extension to randomized crossover trials [10]. The Institutional Review Board of the Sheikh Shakhbout Medical City (SSMC) approved the study protocol (Protocol number MAFREC-271), and all children or their parents/guardians provided written informed consent or assent for study participation.

### 2.1. Trial Design

**Figure 1:**
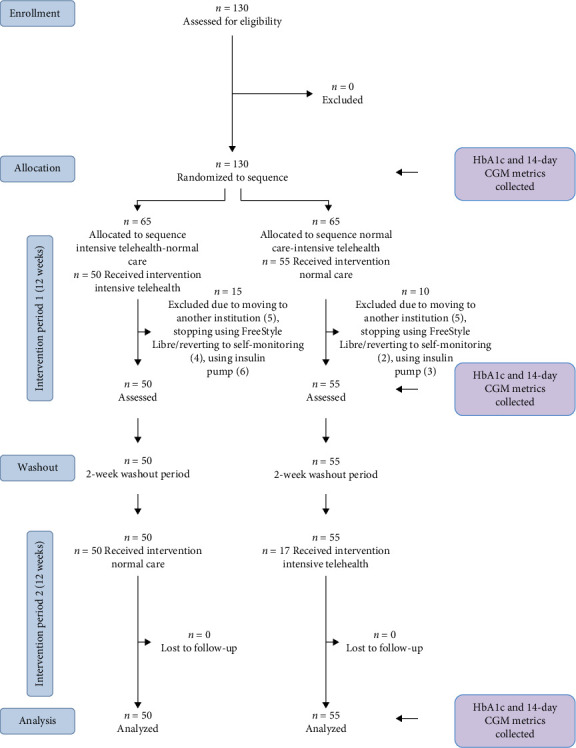
CONSORT flow diagram for crossover trials and study design. TC, teleconsultation intervention; NC, normal care (CGM without teleconsultation).

Patients were enrolled from May 2023 to Nov 2023, with the last follow-up visit occurring in June 2024. This was a randomized, controlled, crossover study of 28 weeks' duration consisting of randomization and run-in (2 weeks), follow-up period one (12 weeks), washout (2 weeks), and follow-up period two (12 weeks) (flow diagram and study overview in Figure 1). Patients were randomly assigned to intensive telehealth intervention follow-up over 12 weeks in treatment period one, followed by CGM with routine clinical care and no intensive telehealth intervention over 12 weeks in treatment period two, or vice versa.

A crossover design was chosen for this study instead of the more traditional randomized, parallel-group design because the within-patient variation was less than the between-patient variation (such as that caused by some patients having a better attitude towards diabetes management regardless of healthcare practitioner [HCP] effort) and thus required fewer patients. In addition, some of the known disadvantages of the crossover design (e.g., larger dropout rate and instability of the patient's condition) were not expected in this study.

Each follow-up period was separated by a 2-week washout. Recognizing that many elements of the intervention were delivered by HCPs and therefore unlikely to carry over, this period was selected for washout, as the default report period for the device is 2 weeks, making it easier to gather data for the correct study period. With respect to potential carry over, the intervention contained an educational component by design that we hoped would persist. However, we were also aware that adolescents often struggle to maintain improved glycemic control once intensive support is withdrawn [11–14]. We therefore took the pragmatic approach that carryover would not preclude the adoption of a crossover trial design.

### 2.2. Participants

The inclusion criteria were (i) patients aged ≤18 years; (ii) diagnosed with type 1 diabetes for at least 6 months; (iii) not meeting individualized HbA1c goals and HbA1c higher than target (i.e., ≥6.5%; 48 mmol/mol) according to 2022 International Society for Pediatric and Adolescent Diabetes (ISPAD) clinical practice consensus guidelines for glycemic targets and glucose monitoring for children and adolescents with diabetes [15]; (iv) taking MDI insulin; (v) attending the SSMC diabetes clinic for regular follow-up; and (vi) using FreeStyle Libre 2 at the start of the study. The exclusion criteria were patients (i) on insulin pump therapy; and (ii) on GCM other than FreeStyle Libre 2, to reduce the influence of known variability between different sensors [16].

### 2.3. Settings and Location

Pediatric Endocrinology Division clinics of SSMC. HbA1c was measured in the SSMC laboratories using a turbidimetric inhibition immunoassay (NGSP/DCCT-aligned) according to standard operating procedures.

### 2.4. Interventions

The intensive telehealth intervention (TC) was intensified follow-up with a weekly teleconsultation and digital support. The weekly teleconsultation session, conducted by telephone, lasted about 20 min and was administered by a trained diabetes educator, who provided support to improve diabetes control. The nominated point of contact for the intervention was the carer, although usually both the patient and the carer joined the meeting, and the session was run with both patients and carers. All structured education was delivered tailored to individual language/level of education needs and in an age-appropriate manner when the patient attended. The structured intervention included eight items delivered each week as follows:

1. Encouragement of adherence to using the CGM device. This included, as necessary, advice on interpreting trend arrows and action to take in response to them, as per our previous publication [17];
2. Troubleshooting connectivity issues with the mobile device;
3. Action to take in response to abnormal glucose readings (both hyperglycemia and hypoglycemia);
4. Revision of insulin adjustments based on physician advice from the latest clinic visit;
5. Education on prevention and management of hypoglycemia;
6. Providing a reminder about upcoming clinic appointments and helping with booking new appointments;
7. Ensuring availability of CGM sensor supplies;
8. Digital support consisted of a thorough review of records in LibreView. During the teleconsultation, the educator accessed the patient's glucose report through the LibreView clinic platform to prompt real-time discussion of the data and to discuss specific episodes of concern.

These items were written on a checklist to ensure that each element was covered during the appointment. If individuals already used carbohydrate counting, this was consolidated in the sessions, but no new education was provided to those not carbohydrate counting.

Normal care (NC) consisted of routine clinic follow-up as per outpatient booking schedules.

### 2.5. Outcomes

The primary outcomes were effects of the teleconsultation intervention on HbA1c % and 14-day GCM metrics of time in range (TIR) %, time above range (TAR) %, time below range (TBR) %, average glucose (mmol/L), glucose variability %, glucose management indicator (GMI) %, average duration of hypoglycemia (min), time sensor active %, and frequency of low glucose events (<70 mg/dL). Targets were > 70% between 70–180 mg/dl (TIR), <4% <70 mg/dl, <1% <54 mg/dl, <25% >180 mg/dl, and <5% >250 mg/dl, as per ISPAD Clinical Practice Consensus Guidelines 2022 [15]. These variables were measured prior to randomization and at the end of each follow-up period.

### 2.6. Sample Size

We hypothesized that the percentage of TIR readings in participants using the education and telemedicine would be at least 6% higher than in the control group [18]. The SD of the per-protocol control group A SD of 10.4 was used for power calculations [19]. Sample size calculations were performed in WINPEPI [20]. Based on a significant improvement in TIR of 6%, assuming an SD of the change in TIR of 10.4, for a power of 80% and *α* = 0.05 with an expected 10% dropout, we aimed to enroll a minimum of 106 patients. As the effect of CGM reported by the Juvenile Diabetes Research Foundation CGM Study Group [18] and Nimri et al. [19] may not be directly relevant to the current study, we also performed a post hoc sample size calculation based on data describing the effects of a virtual education camp on CGM metrics [21]. Based on a significant improvement in TIR of 11.0%, assuming an SD of the change in TIR of 23.0, for a power of 80% and *α* = 0.05 with an expected 10% dropout, a minimum of 39 patients was required.

### 2.7. Randomization

The online GraphPad tool was used (https://www.graphpad.com/quickcalcs/randomize1/) for randomization. After a 2-week “run-in” of usual care, eligible patients were randomly assigned, according to a computer-generated allocation schedule, to one of the two follow-up sequences. After a 2-week washout, patients crossed over to the other treatment. Eligible subjects were randomized in a 1:1 allocation to one of two treatment sequences—intensive telehealth intervention follow-up over 12 weeks in treatment period one, followed by CGM with routine clinical care and no intensive telehealth intervention follow-up over 12 weeks in treatment period two, or vice versa—and received each follow-up for 12 weeks.

### 2.8. Statistical Methods

Data were analyzed in SPSS v29 (IBM Statistics, Armonk, NY). Normality of data was tested with the Shapiro–Wilk test. Continuous variables are described as mean (SD) and categorical variables as counts (%). Cross-over analyses for CGM metrics averaged the between-follow-up difference for each patient within each sequence (expressed as mean [SD]) and then across both sequences, providing an estimate of treatment effect with 95% CIs. The null hypothesis of no within-participant difference was examined with paired *t*-tests. A supplementary carry over analysis was also performed using a linear mixed-effects model including fixed effects for treatment, period, and a carryover term (coded for participants who had received telehealth in period 1), with a random intercept for participant. A *p*-value < 0.05 was considered significant.

## 3. Results

A flow diagram of participants and study overview is shown in Figure 1. Patients were recruited between May and November 2023. After randomization, 25 patients were excluded due to changing to an insulin pump, moving for treatment at another institution, or stopping using FreeStyle Libre, either moving back to self-monitoring blood glucose or another type of CGM.

The baseline demographic and CGM characteristics for each sequence group and for all patients are shown in Table 1. The average age of the analyzed study cohort (*n* = 105) was 11.8 (4.2) years (range 2–18 years), and 48.6% were female. The average duration of diabetes was 3.5 (3.0) years (range 6 months to 11.9 years). Overall, with reference to 2022 ISPAD clinical practice consensus guidelines for glycemic targets and glucose monitoring for children and adolescents with diabetes [15], the study population had characteristics of suboptimal diabetes control prior to study commencement, with an average HbA1c of 9.4 (1.6) % (79 (13) mmol/mol; target < 6.5%) and 14-day CGM metrics of TIR 51.4 (16.7) % (target >70% between 3.9 and 10 mmol/L), TAR 46.1 (16.9) % (target <25%: >10 mmol/L), and glycemic variability 40.9 (6.6) %CV (target ≤36%). TBR was within target range at baseline 2.9 (3.3) % (target <4%: <3.9 mmol/L).

Fifty participants were analyzed in the intensive telehealth-NC sequence and 55 participants were analyzed in the no NC-intensive telehealth sequence. The analysis was performed according to the assigned groups.

The estimated treatment effect sizes based on within-individual differences, along with results according to each sequence, are shown in Table 2. The precision (95% CIs) of the estimated effect of intensive telehealth vs. NC during follow-up are also shown, along with hypothesis testing of the within-participant difference (paired sample *t*-test).

**Figure 2:**
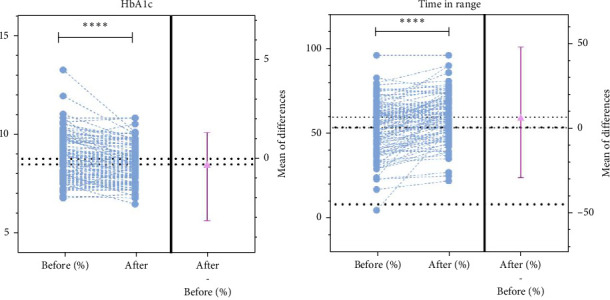
Treatment effect sizes of intensive telehealth on HbA1c (left) and time in range (right). *⁣*^*∗∗∗∗*^ denotes *p*  < 0.0001, paired *t*-test.

Compared with NC during follow-up, intensified follow-up with weekly teleconsultation and digital support was associated with a decrease in HbA1c (−0.29 (0.60) %, 95% CIs −0.41 to −0.17, *p*  < 0.001) and an increase in TIR (by 6.2 (11.0) %, 95% CIs 4.0–8.3, *p*  < 0.001; Figure 2). Furthermore, intensive telehealth was associated with a decrease in TAR (by 5.5 (11.6) %, 95% CIs −7.7 to −3.2, *p*  < 0.001), decrease in average glucose level (by 0.73 (1.59) mmol/L, 95% CIs −1.0 to −0.42, *p*  < 0.001), decrease in glucose variability (by 1.25 (3.6) %CV, 95% CIs −1.96 to −0.55, *p* < 0.001), decrease in GMI (by 0.20 (0.57) %, 95% CIs −0.31 to −0.09, *p*  < 0.001), and decrease in frequency of low glucose events (<70 mg/dL) (by 0.12 (0.47) events, 95% CIs −0.22 to −0.03, *p*=0.009) (Table 2). Intensive telehealth did not have an effect on TBR, which was already within target in this population, including when split into low and very low glucose levels (*p*=0.206 and *p*=0.644, respectively).

To explore potential sequence and carry over effects, we examined outcomes by randomization group. HbA1c and TIR at baseline, 12 weeks, and 26 weeks separately for the telehealth-first and routine care-first sequences are shown in Table S1. Participants randomized to telehealth first improved at 12 weeks but tended to lose benefit after returning to routine care, whereas those randomized to routine care first improved when they subsequently received the intervention. In period 2 comparisons, HbA1c and TIR differed significantly between sequences (Table S2), consistent with a carryover effect. A mixed-effects model with an explicit carry over term similarly indicated carry over for HbA1c but not for TIR.

In terms of intervention delivery, teleconsultations were performed uniformly for all participants as per protocol, with deferral of the consultation for a maximum of 48 h in few cases when families were not available for the call. No patient reported any harms or negative consequences of intensified follow-up with weekly teleconsultation and digital support.

## 4. Discussion

Improving glycemic control in children and adolescents with type 1 diabetes using CGM requires frequent contact with HCPs and data review. This is especially true for CGM, which requires skills and education to understand the abundant and varied data that influence insulin therapy [22]. Here we examined the impact of adding an intensive telehealth intervention to CGM use on glycemic control in children and adolescents with type 1 diabetes taking MDI insulin. Using a randomized crossover trial design, we show that intensified follow-up with weekly teleconsultation, tele-education, and digital support was associated with small but significant and positive impacts on most glycemic control metrics: a decrease in HbA1c, increase in TIR, a decrease in TAR, a decrease in average glucose levels, a decrease in glucose variability, a decrease in GMI, and a decrease in the frequency of low glucose events. The small but statistically significant reduction in HbA1c (by 0.29%) and an increase in TIR (6.2%) over 12 weeks is comparable to the effect observed in clinical trials when GCM is compared with standard blood glucose monitoring [23]. While the clinical significance of these findings requires prospective evaluation with respect to clinical outcomes over time, the intervention offers the potential for clinical impact. The intervention shifted the HbA1c and TIR distribution in the right direction to address the challenge of achieving glycemic control in children and adolescents [15]. Our study therefore has important implications for the way in which children and adolescents on CGM should be followed up in routine practice.

Telemedicine encompasses a range of different technologies (telephone, text messaging, computer software, videoconferencing, and smartphone apps [24]) and approaches including telemonitoring (i.e., automated remote health monitoring of pathophysiological data [25]), tele-education (the application of remote communication technologies to deliver educational content [26]), and teleconsultations (interactions with HCPs via remote communication technologies [27]). Telemedicine has been applied in its various forms to the management of children and adolescents with diabetes for over 20 years. A recent meta-analysis of 20 RCTs studying diverse telemedicine interventions on glycemic control in children and adolescents with type 1 diabetes reported that, overall, telemedicine use was associated with a significant reduction in HbA1c levels [24]. Reflecting the heterogeneity of the included studies, subgroup analysis suggested that younger children, use of smartphone apps, interventions that included medication dose adjustments, and “complete” telemedicine interventions (i.e., no face-to-face contact) reported significantly larger effects of telemedicine [24]. However, few studies have specifically and directly examined the value of teleconsultation in young people on CGM. In the Virtual Outpatient Diabetes Clinic for Children and Youth (VIDIKI) study and its extension [11, 12], children and adolescents with type 1 diabetes using a CGM were assigned to receive either regular (3-month) follow-up or monthly video consultations in addition to regular care, finding that the intervention was associated with significant and persistent decreases in HbA1c compared with controls [12]. Furthermore, the intervention was associated with increased treatment satisfaction in parents and a decrease in their diabetes-specific burden and distress [12]. Although mainly examining youth with new-onset diabetes, the 4T Study (teamwork, targets, technology, and tight control) also shared some similarities with our intervention, as in addition to initiating CGM in the new-onset period, a subset of participants also participated in remote monitoring (CGM data reviewed by a certified diabetes care and education specialist and dose adjustments recommended using secure messaging). Although not directly comparable with our cohort in terms of population or intervention, HbA1c was approximately 0.14% to 0.18% lower in the 4T with remote monitoring group than those with remote monitoring at various time points, again favoring a small but significant benefit for active remote intervention [28].

As there is no standard, agreed protocol for telehealth delivery for children and adolescents on CGM, we took an evidenced-based approach to design an integrated intervention that included major elements of a complete telehealth intervention (teleconsultation, tele-education, telecase management, and telemonitoring) that have been shown to positively impact glycemic metrics in children and adolescents on CGM. We also aimed to use the latest available telediabetology solutions for observation, documentation, and intervention [29]. First, the intervention was built on a platform of teleconsultation by telephone, as several meta-analyses have shown a favorable impact of telemedicine, especially teleconsultation alone [24, 30], over usual care/face-to-face contact on HbA1c levels in both children/adolescents [24, 30] and adults [31]. Second, we provided individualized assessments and telecase management that included insulin adjustments, because individualized telehealth assessments (i.e., where the patient is assessed individually and the intervention is tailored to the assessment) targeted at improving knowledge have been shown to have a greater treatment effect [24, 30]. Third, we incorporated a tele-education component (education on prevention and management of hypoglycemia and skill building, such as action to take in response to abnormal glucose readings or trend arrows and real-time discussion of the data to discuss specific episodes of concern), as not only has tele-education been shown to have a positive effect on glycemic control [30] in the general population of children and adolescents with type 1 diabetes, but virtual education has also been shown to be especially useful in children with diabetes using advanced technologies like CGM and closed-loop systems [21, 32]. Fourth, the use of CGM lent itself to a telemonitoring approach through the upload of patient data to the clinical platform for HCP review prior to consultations.

Finally, we favored an intensive program, as high-intensity (weekly) telemedicine interventions appear to have a greater magnitude of effect on reducing HbA1c levels than those that occur less frequently, both in meta-analysis [−0.24 (−0.49−0.01) at least once weekly vs. −0.09 (−0.23−0.06) for less frequently [12]] and randomized controlled settings [13]. Furthermore, the VIDIKI study showed that a higher frequency of contact led to short-term therapy adjustments and an increase in the ability to adjust therapy independently, together with improvements in important patient/carer reported satisfaction outcomes [12, 33, 34].

Our finding that the intervention had no effect on TBR is consistent with findings from other studies examining CGM that the treatment modality (MDI or insulin pump) is associated with TIR and TAR but not TBR [7]. The reasons for this are unclear. In our cohort, while other glycemic metrics were well out of range, TBR was within range for the study population. Apart from potentially masking small magnitude effects of the intervention, this also reflects our local experience that fear of hypoglycemia (FoH) [35] is especially high in our practice in the UAE. We observe that parents (most often mothers) have a major FoH and tend to underestimate their children's insulin needs. Despite intensive education on the harmful effects of both, the risk of serious hypoglycemia-related side effects such as brain damage seems to override other harms, and we commonly see mothers giving extra snacks or meals before bed (over-compensatory behaviors) purely to avoid hypoglycemia, even if pre-bed readings are normal or borderline high. Clinicians must have a high index of suspicion for FoH, perhaps through routine use of screening tools such as the Hypoglycemia Fear Survey (—Parents) or other instruments, as recommended recently [36].

## 5. Strengths and Limitations

There have been few prospective, controlled studies of the impact of telemedicine interventions on children and adolescents with type 1 diabetes on CGM, and this study provides robust efficacy data on a new telemedicine intervention with measurable impact on many important glycemic control endpoints in a difficult-to-manage population. Our application of a crossover trial design, which eliminates between-subject variability, and reported according to CONSORT standards provides confidence in the results. We provide CIs so that the data can be included in future meta-analyses [37]. The integrated intervention encompassed all the major aspects of telemedicine including teleconsultation, tele-education, telecase management, and telemonitoring that should be readily applicable in specialist diabetic clinics by multidisciplinary personnel.

A major limitation of any crossover trial is carryover effects [38], where the previous treatment may bias the treatment effects observed in the second sequence. Although we reasoned that many elements of the intervention were delivered by HCPs (dose adjustments, reminders, technical support) and therefore unlikely to carry over, the intervention also contained an educational component by design that we would hope would persist. Our supplementary analyses demonstrated evidence of carry over, particularly for HbA1c, with partial persistence of benefits from the educational component. Nevertheless, the primary crossover analysis remains valid, as the CONSORT extension cautions against discarding second-period data [10], which can bias estimates [38, 39]. The data are consistent with the observation that glycemic improvements achieved during intensive support are difficult to sustain once support is withdrawn, reinforcing the importance of ongoing structured telehealth support for durability of effect. This is perhaps not surprising in this age group, who are known to struggle to sustain improved control after intensive diabetes interventions [13]. It is likely that such an intervention, if implemented in routine practice, would need to be continued over the longer term or perhaps indefinitely, and future studies should examine efficacy over an extended period beyond the 12 weeks examined here to establish durability of response. The lack of persistence also raises the issue of feasibility of implementation in clinical practice. However, while provision of resources for intensive management can be challenging, the aim of this study was to provide evidence that provision of these resources might be warranted based on efficacy, and our study provides a reference to calculate cost-effectiveness in clinical business cases and future clinical trials.

We also noted a decrease in HbA1c and an increase in sensor wear time from randomization (prior to entering into the crossover period) to the end of intervention period 1 for both the telehealth and routine care groups; an increase in sensor wear time could have had a significant impact on glycemic outcomes. This observation suggests that trial participation and starting structured follow-up alone were associated with improvements in both glycemic parameters and CGM adherence, possibly due to the trial participation (Hawthorne) effect, where increased motivation, adherence, contact, and monitoring when under study conditions have a positive effect on outcomes [40]. However, by adopting a crossover design, we were able to determine crossover-adjusted differences between telehealth and routine care to isolate the benefit of telehealth beyond general trial effects, which is more likely to reflect the magnitude of effect in practice.

We also limited our population to individuals on CGM taking MDI insulin, and it would be useful to understand efficacy in children and adolescents using newer HCL systems. As we did not record total daily insulin requirements, C-peptide levels, nor body weights of participants as part of the protocol, we were unable to assess these metrics to provide further insights into the metabolic impact of the intervention or insulin reserve. Participants were supposed to wear the CGM sensor all the time, and we did not collect data during the period when the sensor was not active, as it was not part of the protocol, could not be predicted prospectively, and the objective was to utilize CGM data only. However, we acknowledge that this is a limitation, especially given that one sequence group had a mean sensor active time of 57.8% during the run-in period, probably contributing to their failure to meet individualized HbA1c goals. Future studies should account for this potential limitation, especially in populations with suboptimally controlled diabetes who may be less likely to be CGM-adherent. Furthermore, we did not examine any patient-reported or quality-of-life outcomes, such as diabetes-related stress, and it would be interesting in future implementations of the intervention to examine its impact on psychological wellbeing. Finally, we did not perform any health economic analysis, which is necessary for real-world implementation.

## 6. Conclusions

This randomized, controlled, and crossover study shows that intensified follow-up with weekly teleconsultation and digital support results in a small but significant improvement in glycemic control metrics in the challenging population of children and adolescents with type 1 diabetes.

## Figures and Tables

**Table 1:** Demographic and CGM characteristics for each sequence group prior to the start of the study.

Variable	Intensive telehealth-normal care (*n* = 50)	Normal care-intensive telehealth (*n* = 55)	Total (*n* = 105)
Age, years (SD)	12.4 (3.7)	11.1 (4.6)	11.8 (4.2)
Sex, *n* (%)			
Male	23 (46)	31 (56.4)	54 (51.4)
Female	27 (54)	24 (43.6)	51 (48.6)
Duration of diabetes, years (SD)	4.2 (3.2)	3.5 (2.9)	3.5 (3.0)
HbA1c, % (SD)	9.7 (1.8)	9.0 (1.3)	9.4 (1.6)
HbA1c, mmol/mol (SD)	83 (15)	75 (11)	79 (13)
Time in range, % (SD)	51.5 (15.2)	52.1 (2.5)	51.5 (16.7)
Time above range, % (SD)	46.3 (15.0)	45.4 (18.7)	46.1 (16.9)
Time below range, % (SD)	2.3 (2.3)	3.6 (4.0)	2.9 (3.3)
Average glucose, mmol/L (SD)	12.2 (3.0)	11.4 (2.9)	11.8 (2.9)
Glucose variability, % CV (SD)	40.4 (6.1)	41.7 (7.0)	40.9 (6.6)
Glucose management indicator, % (SD)	8.5 (1.2)	8.2 (1.1)	8.4 (1.2)
Average duration of hypoglycemia, min (SD)	77.6 (48.4)	91.9 (62.7)	83.2 (56.6)
Time sensor active, % (SD)	57.8 (20.8)	71.9 (19.1)	64.2 (21.3)
Frequency of low glucose events (<70 mg/dl), *n* (SD)	0.67 (0.85)	0.17 (0.43)	0.4 (0.7)

**Table 2:** Effects of intensified follow up with weekly teleconsultation and digital support compared with normal care according to intervention period and sequence.

Variable	Intervention sequence	Intervention period 1	Intervention period 2	Within-individual differences: (A–B)
HbA1c, % (SD)	Intensive telehealth-normal care			
	Mean (SD)	8.7 (1.0)	8.9 (1.2)	−0.19 (0.59)
	Sample size	50	50	50
	Normal care-intensive telehealth			
	Mean (SD)	8.5 (1.1)	8.2 (0.8)	−0.37 (0.62)
	Sample size	55	55	55
	Treatment effect			
	Mean (SD)			−0.29 (0.60)
	95% confidence intervals			−0.41 to -0.17
	Sample size			105
	*t*-test for paired samples			<0.001

Time in range, % (SD)	Intensive telehealth-normal care			
	Mean (SD)	58.1 (14.2)	51.0 (15.6)	7.2 (10.4)
	Sample size	50	50	50
	Normal care-intensive telehealth			
	Mean (SD)	54.6 (15.4)	59.7 (13.2)	5.1 (11.8)
	Sample size	55	55	55
	Treatment effect			
	Mean (SD)			6.2 (11.0)
	95% confidence intervals			4.0 to 8.3
	Sample size			105
	*t*-test for paired samples			<0.001

Time above range, % (SD)	Intensive telehealth-normal care			
	Mean (SD)	38.9 (13.9)	45.8 (15.2)	−6.9 (10.1)
	Sample size	50	50	50
	Normal care-intensive telehealth			
	Mean (SD)	43.2 (16.2)	39.1 (14.1)	-3.8 (12.8)
	Sample size	55	55	55
	Treatment effect			
	Mean (SD)			−5.5 (11.6)
	95% confidence intervals			−7.7 to -3.2
	Sample size			105
	*t*-test for paired samples			<0.001

Time below range, % (SD)	Intensive telehealth-normal care			
	Mean (SD)	2.8 (2.7)	2.7 (2.6)	0.02 (1.87)
	Sample size	50	50	50
	Normal care-intensive telehealth			
	Mean (SD)	3.1 (4.3)	2.6 (3.6)	−0.48 (2.9)
	Sample size	55	55	55
	Treatment effect			
	Mean (SD)			−0.21 (2.4)
	95% confidence intervals			−0.68 to 0.26
	Sample size			105
	*t*-test for paired samples			0.379

Average glucose, mmol/L (SD)	Intensive telehealth-normal care			
	Mean (SD)	11.6 (2.7)	11.9 (2.5)	−0.24 (1.5)
	Sample size	50	50	50
	Normal care-intensive telehealth			
	Mean (SD)	11.1 (2.8)	9.9 (2.4)	−1.18 (1.6)
	Sample size	55	55	55
	Treatment effect			
	Mean (SD)			−0.73 (1.59)
	95% confidence intervals			−1.0 to -0.42
	Sample size			105
	*t*-test for paired samples			<0.001
Glucose variability, %CV (SD)	Intensive telehealth-normal care			
	Mean (SD)	37.5 (4.7)	38.1 (5.5)	−0.60 (3.3)
	Sample size	50	50	50
	Normal care-intensive telehealth			
	Mean (SD)	39.8 (6.2)	37.8 (5.4)	−1.8 (3.7)
	Sample size	55	55	55
	Treatment effect			
	Mean (SD)			−1.25 (3.6)
	95% confidence intervals			−1.96 to -0.55
	Sample size			105
	*t*-test for paired samples			<0.001

Glucose management indicator, % (SD)	Intensive telehealth-normal care			
	Mean (SD)	8.4 (1.1)	8.5 (1.2)	−0.11 (0.45)
	Sample size	50	50	50
	Normal care-intensive telehealth			
	Mean (SD)	8.0 (1.0)	7.7 (0.8)	−0.26 (0.66)
	Sample size	55	55	55
	Treatment effect			
	Mean (SD)			−0.20 (0.57)
	95% confidence intervals			−0.31 to -0.09
	Sample size			105
	*t*-test for paired samples			<0.001

Average duration of hypoglycemia, min (SD)	Intensive telehealth-normal care			
	Mean (SD)	83.5 (49.3)	85.1 (48.4)	−1.64 (38.1)
	Sample size	50	50	50
	Normal care-intensive telehealth			
	Mean (SD)	63.7 (42.8)	66.6 (41.2)	2.87 (31.9)
	Sample size	55	55	55
	Treatment effect			
	Mean (SD)			0.53 (34.7)
	95% confidence intervals			−6.2 to 7.2
	Sample size			105
	*t*-test for paired samples			0.875

Time sensor active, % (SD)	Intensive telehealth-normal care			
	Mean (SD)	69.5 (17.2)	70.7 (17.1)	−1.2 (12.7)
	Sample size	50	50	50
	Normal care-intensive telehealth			
	Mean (SD)	76.4 (16.7)	80.7 (13.6)	4.2 (9.7)
	Sample size	55	55	55
	Treatment effect			
	Mean (SD)			1.39 (12.6)
	95% confidence intervals			−1.04 to 3.82
	Sample size			105
	*t*-test for paired samples			0.259

Frequency of low glucose events (<70 mg/dl), *n* (SD)	Intensive telehealth-normal care			
	Mean (SD)	0.14 (0.41)	0.26 (0.49)	−0.12 (0.52)
	Sample size	50	50	50
	Normal care-intensive telehealth			
	Mean (SD)	0.17 (0.38)	0.04 (0.19)	−0.13 (0.44)
	Sample size	55	55	55
	Treatment effect			
	Mean (SD)			−0.12 (0.47)
	95% confidence intervals			−0.22 to -0.03
	Sample size			105
	*t*-test for paired samples			0.009

## Data Availability

Raw data are available from the corresponding author upon request.

## Supporting Information

Supporting Information Table S1 Baseline and intervention period values by sequence group. Table S2 Estimates from second period sequence comparison and mixed-effects model including explicit carry over term.
