# Supplementary material for: Effect of an Intensive, Integrated Telehealth Intervention on Glycemic Control in Children and Adolescents With Type 1 Diabetes Using Continuous Glucose Monitoring: A Randomized, Crossover Trial
Source: Pediatr Diabetes. 2025 Sep 27;2025:7261998. doi: 10.1155/pedi/7261998 (PMC12496141; doi:10.1155/pedi/7261998)
Supplement: Supporting Information — Table S1 Baseline and intervention period values by sequence group. Table S2 Estimates from second period sequence comparison and mixed-effects model including explicit carry over term. [file 7261998.f1.docx]

**Supplementary Table 1.** Baseline and intervention period values (mean ± SD) by sequence group

| Variable | Telehealth → Normal care: Baseline (n=50) | Telehealth → Normal care: Period 1 (n=50) | Telehealth → Normal care: Period 2 (n=50) | Normal care → Telehealth: Baseline (n=55) | Normal care → Telehealth: Period 1 (n=55) | Normal care → Telehealth: Period 2 (n=55) |
| --- | --- | --- | --- | --- | --- | --- |
| HbA1c (%) | 9.7 (1.8) | 8.7 (1.0) | 8.9 (1.2) | 9.0 (1.3) | 8.5 (1.1) | 8.2 (0.8) |
| Time in range (%) | 51.5 (15.2) | 58.1 (14.2) | 51.0 (15.6) | 52.1 (2.5) | 54.6 (15.4) | 59.7 (13.2) |
| Time above range (%) | 46.3 (15.0) | 38.9 (13.9) | 45.8 (15.2) | 45.4 (18.7) | 43.2 (16.2) | 39.1 (14.1) |
| Time below range (%) | 2.3 (2.3) | 2.8 (2.7) | 2.7 (2.6) | 3.6 (4.0) | 3.1 (4.3) | 2.6 (3.6) |
| Average glucose (mmol/L) | 12.2 (3.0) | 11.6 (2.7) | 11.9 (2.5) | 11.4 (2.9) | 11.1 (2.8) | 9.9 (2.4) |
| Glucose variability (%CV) | 40.4 (6.1) | 37.5 (4.7) | 38.1 (5.5) | 41.7 (7.0) | 39.8 (6.2) | 37.8 (5.4) |
| Glucose management indicator (%) | 8.5 (1.2) | 8.4 (1.1) | 8.5 (1.2) | 8.2 (1.1) | 8.0 (1.0) | 7.7 (0.8) |
| Average duration of hypoglycemia (min) | 77.6 (48.4) | 83.5 (49.3) | 85.1 (48.4) | 91.9 (62.7) | 63.7 (42.8) | 66.6 (41.2) |
| Time sensor active (%) | 57.8 (20.8) | 69.5 (17.2) | 70.7 (17.1) | 71.9 (19.1) | 76.4 (16.7) | 80.7 (13.6) |
| Frequency of low glucose events (<70 mg/dl) | 0.67 (0.85) | 0.14 (0.41) | 0.26 (0.49) | 0.17 (0.43) | 0.17 (0.38) | 0.04 (0.19) |

**Supplementary Table 2.** Estimates from second period sequence comparison and mixed-effects model including explicit carry-over term. Values are estimated differences with 95% CIs and p-values.

| **Endpoint** | **Method** | **Estimate** | **95% CI** | **p-value** |
| --- | --- | --- | --- | --- |
| HbA1c (%) | Period 2 comparison (AB vs BA) | –0.68 | –1.06 to –0.29 | 0.0007 |
| TIR (%) | Period 2 comparison (AB vs BA) | +8.76 | +3.20 to +14.33 | 0.0023 |
| HbA1c (%) | Mixed model (carry-over term) | +0.78 | 0.04 to 1.53 | 0.0399 |
| TIR (%) | Mixed model (carry-over term) | –5.09 | –15.25 to 5.08 | 0.3267 |

AB = telehealth first then routine care; BA = routine care first then telehealth. The period 2 comparison tests for differential carry-over by comparing outcomes at the end of period 2 between sequences (negative values indicate lower outcomes in AB vs BA). Mixed model includes an explicit carry-over term, defined as exposure to telehealth in the prior period, with a random intercept for subject. A significant positive carry-over estimate indicates persistence of intervention effect into the subsequent period.
